# Prior Diagnoses and Age of Diagnosis in Children Later Diagnosed with Autism

**DOI:** 10.1007/s10803-024-06637-3

**Published:** 2024-11-25

**Authors:** Maire C. Diemer, Emily Gerstein

**Affiliations:** 1 Medical University of South Carolina, 171 Ashley Ave, Charleston, SC 29425 USA; 2 University of Missouri- St. Louis, 1 University Blvd, St. Louis, MO 63121 USA

**Keywords:** Autism, Diagnostic load, Black, Medicaid, Race, Gender, Autistic girls, Rural

## Abstract

Awareness of autism is rising, yet social determinants of health impact ages of diagnosis, and diagnostic load. Unequal rates of diagnoses may indicate biases in the healthcare system. This study investigates six prior diagnoses (ADHD, conduct, adjustment, anxiety, mood, and intellectual disability) assigned to children who are later diagnosed with autism. The study investigates how race, sex, and geographic factors were associated with age of diagnosis and diagnostic load. A sample of 13,850 (78.16% male and 14.43% Black, with 57.95% of children living in urban regions) children aged 2–10 who were diagnosed with autism on Missouri Medicaid between 2015 and 2019 were studied. Indicated that being White, living urban, and having more prior diagnoses were associated with older age of autism diagnosis. Using logistic regressions, being White was associated with a child being more likely diagnosed with all prior diagnoses aside from intellectual disability. Being male was related to a higher likelihood of ADHD, and lower likelihood of intellectual disability. Being White was associated with higher likelihood of most diagnoses, even in urban-only samples, potentially reflecting more access to providers and office visits. Living in rural areas was also associated with earlier diagnosis and more prior diagnoses such as ADHD and conduct, which may be due to types of providers or specialists seen. Future research should look at barriers to diagnosis and the advantages and disadvantages of a higher diagnostic load.

**Supplementary Information:**

The online version contains supplementary material available at 10.1007/s10803-024-06637-3.

Autism is a complex neurodevelopmental disability involving difficulties with social skills and communication, as well as behavioral inflexibility, with high rates of psychiatric and medical comorbidities (American Psychological Association (APA), 2013). Autism can be reliably diagnosed around 2 years old, but the average age in the United States (US) is 4–5 years old, and diagnostic rates have significantly risen in the past 20 years (Maenner et al., 2023; Zwaigenbaum & Penner, 2018). Unfortunately, disparities persist. Historically, Black children were diagnosed less frequently and are still diagnosed later than non-Hispanic White children (Fombonne & Zuckerman, 2022; Jo et al., 2015). Females are diagnosed less frequently and later (McDonnell et al., 2020), and rural regions lag urban regions. Similarities and co-occurrence of symptoms between autism and other common early childhood disorders may lead to difficulty in diagnosis (Mandell et al., 2007). Medical and psychiatric complexity can make autism diagnosis more complicated and enhance the impact of bias and disparities. This study investigates diagnostic pathways and lingering inequities by examining how race, gender, and geography are associated with age of autism diagnosis and comorbid diagnoses assigned prior to autism.

## Diagnostic Pathways

Age of autism diagnosis autism diagnosis is widely used as a proxy for disparities in access to services (Jo et al., 2015; van ‘t Hof et al., 2020), as it can be diagnosed by age 2 and is not considered to ‘onset,’ at a later age. When a diagnosis of autism is delayed, children may accumulate other diagnoses, which are later more parsimoniously explained by autism (Fusar-Poli et al., 2020; Mazefsky et al., 2012). More diagnoses can mean higher prescription dosages and loads, causing challenges with medication management, compliance, side effects, and more (Espadas et al., 2020; Houghton et al., 2017). Sometimes delays come at the point of initial screening, completed by a pediatrician, Bellesheim et al. (2020) report that many, particularly rural, pediatricians do not screen in line with American Academy of Pediatrics recommendations.

Additionally, delayed diagnosis means children and families can lose years of early interventions which might support independent functioning (Farmer et al., 2014). Indeed, access to formal educational support plans rely on disability diagnoses and may be punitive when not designed for autistic children (Slaughter et al., 2019). Lower socioeconomic status (SES) and higher IQ are both related to later age of diagnosis (Mazurek et al., 2014), thus, certain children may have a better chance of receiving early and appropriate support services.

### Prior Diagnoses

Autistic people are at increased likelihood for medical and psychiatric co-occurring conditions (Rosen et al., 2018), and more research is needed to understand the ways that this impacts diverse autistic populations, and age of autism diagnosis. Given the commonality of co-occurring diagnoses, Mandell et al. (2007) published a study using Medicaid data to examine diagnoses children received before autism. The data included children living in urban Philadelphia in the late 1990s, who were diagnosed with Autistic Disorder in the DSM-IV (APA, 1998). More than 56% of the children received a diagnosis other than autism on their first mental health visit, most commonly ADHD. Race impacted outcomes; Black children were five times more likely to receive a diagnosis of adjustment disorder compared to ADHD, and more than 2.5 times more likely to receive a diagnosis of Conduct Disorder (Mandell et al., 2007). Boys were also more likely than girls to receive conduct disorder. Possible explanations authors suggested included symptom presentation, parent report, or clinician bias (Mandell et al., 2007). Research with more recent data points that includes a more expansive examination of gender, and geographic region would improve the field’s understanding of these trends. This paper focuses on updated data (20 years after Mandell), and can capture new trends in race, gender, age, and criterion of spectrum diagnosis, as well as neurodevelopmental concerns such as attention deficit/hyperactivity disorder (ADHD) and oppositional defiant disorder (ODD), mood and anxiety diagnoses, adjustment disorders, intellectual disability, as these are commonly diagnosed in childhood and co-occurring with autism.

Significant research has investigated the symptomatic and genetic/ environmental risk factor overlap among ADHD, ODD, conduct disorder (CD), and autism (Geluk et al., 2012). Co-occurrence between autism and ADHD is estimated at approximately 30% (Rosen et al., 2018). Symptoms of inattention, such as lack of eye contact, or fine motor stereotypy (which can be viewed as fidgeting), can be particularly difficult to differentiate between the two disorders. Positive correlations among autism, ADHD, ODD, and CD symptoms have been found in both boys and girls (Kerekes et al., 2014).

Anxiety is considered the most common co-occurring diagnosis with autism (Lai et al., 2019). This may present as social anxiety, generalized anxiety, or may be environmentally determined (e.g., related to sensory concerns or routine disruptions). Little research exists on adjustment disorders, and their differentiation from healthy or typical reactions to stress are unclear (Casey & Bailey, 2011). Adjustment diagnoses are commonly used to secure referrals for other services, when clinicians are not comfortable making a diagnosis (Chomienne et al., 2011). Relatedly, researchers have also found support for a connection between autism and obsessive–compulsive disorder (OCD), particularly in restricted and repetitive behaviors and preferences for routines, which can be diagnostic across both of these labels (Jiujias et al., 2017; Pazuniak & Pekrul, 2020). There is also evidence for shared genetic and etiological origin (Meier et al., 2015).

### Inequities in Autism Research Participant Samples

An intersectional approach to research in autism elucidates the ways in which race, biological sex, geographical location, cognitive ability, and many other factors combine to impact the lives of diverse autistic people. Autistic Black children are diagnosed later, despite parents reporting concerns at similar times to their White counterparts (Constantino et al., 2020). The intersectional identity as both Black and disabled means research indicates increased risk of difficulty securing coordinated care, obtaining diagnosis, or feeling mistreated by providers (Bishop-Fitzpatrick & Kind, 2017; Broder-Fingert et al., 2020; Cascio et al., 2020). Furthermore, gold-standard measures for autism evaluation, have also been criticized for their lack of cultural relevance to diverse populations (Kim et al., 2013; (ADI-R; validation sample was 4% Black, 77% male); Pugliese et al., 2015 (ADOS-2; validation sample was 6.4% Black, 77% male); Ronkin et al., 2021).

Females are diagnosed with autism at lower rates than males, approximately 4:1; the ratio is reduced to 2:1 when looking at children with intellectual disability (Hull & Mandy, 2017). The field debates whether this discrepancy is due to a female protective effect, or whether girls are being under-diagnosed due to bias in diagnostic measures (Hull & Mandy, 2017; Kreiser & White, 2014). This study will look at biological sex as a factor in diagnosis and uses female or girl in reference to biological sex at birth.

Families of autistic children living in rural areas report less access to specialized providers, intervention options, and longer waitlists (Antezana et al., 2017; Mello et al., 2016; Zhang et al., 2017). To manage distance-to-care burdens, rural schools can become a primary resource for families, however, this can mean delays in services (Antezana et al., 2017). Living in more rural areas is also related to higher rates of intellectual disability co-occurrence (Palmer et al., 2010). Socioeconomic status can interact with race and rurality to impact families’ abilities to access evaluations, coordinated care, educational supports, early intervention services, and support through adulthood. Research suggests that higher SES is associated with higher rates of autism, and research participation in autism research studies likely due to diagnostic resources (rather than true prevalence differences; Cascio et al., 2021; Durkin et al., 2017).

### Current Study

Delayed diagnoses of autism reveal inequities in access to diagnostic evaluations and healthcare supports. This study examined age at autism diagnosis as well as previous diagnoses received by autistic children enrolled in Missouri Medicaid. The first aim of this study was to examine how age of diagnosis of autism from 2 to 10 years old in Missouri differs based on sociodemographic factors. It was hypothesized that Black children would have a delayed diagnostic age compared to White children, girls would have a delayed diagnostic age compared to boys, children living in rural areas would have a delayed diagnostic age compared to those living in an urban setting, and number of prior diagnoses would be associated with later diagnosis.

The second aim was to examine prior diagnoses assigned to children who later receive autism diagnoses and whether race, sex, or geographic location impact those diagnoses. The diagnostic categories were chosen based on common childhood and developmental diagnoses, common autism comorbidities, and capture a sampling that are diagnosed across genders. It was hypothesized Black children would be more likely to receive prior diagnoses of conduct disorders compared to the larger population, girls would be more likely to receive anxiety- and obsessive–compulsive (OCD) related disorders compared to boys, and children in rural regions would be more likely to receive diagnoses of ADHD and adjustment disorders compared to children in urban settings. The authors would stratify by race, geography, and sex to look at differences in prior diagnoses as an exploratory hypothesis.

## Methods

### Participants

**Fig. 1:**
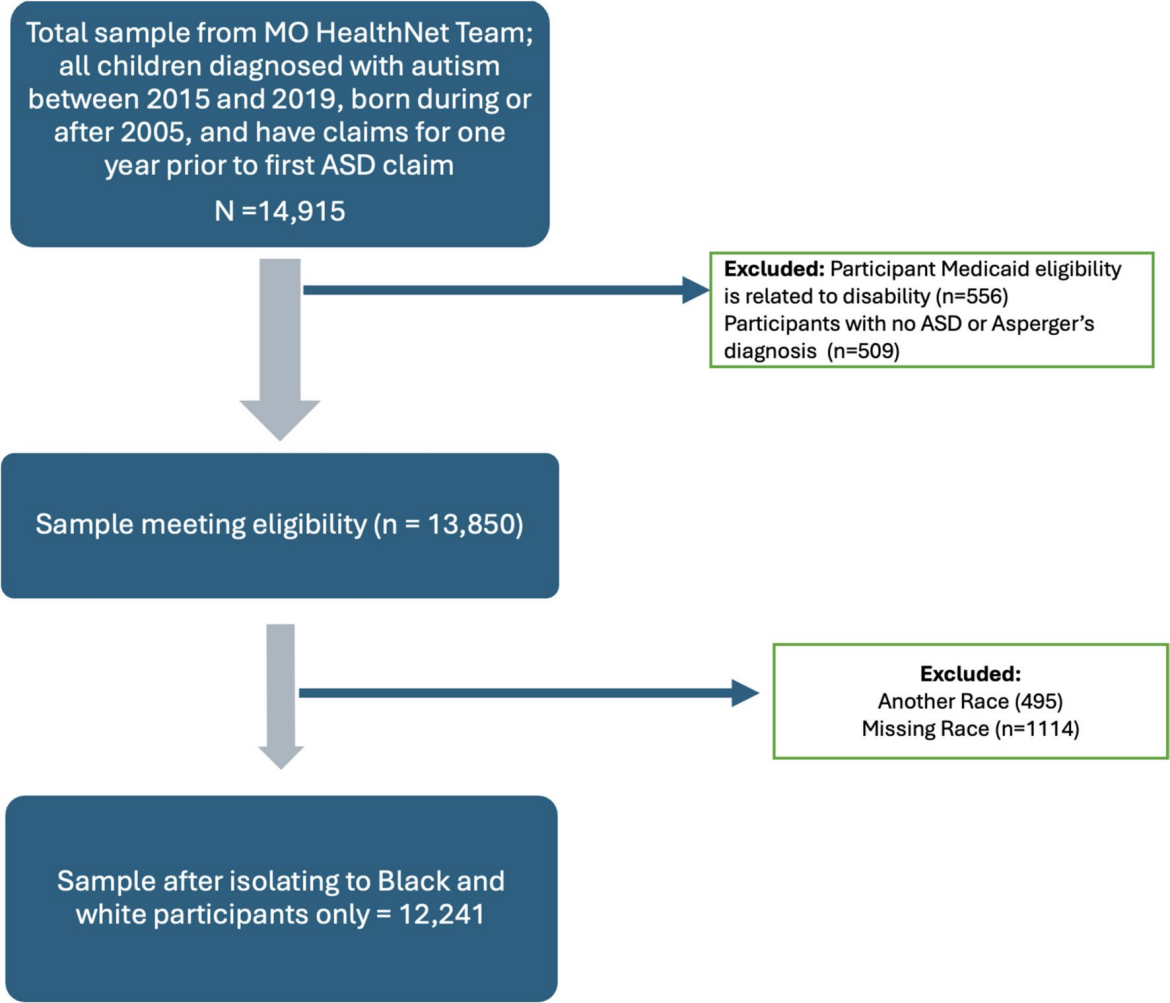
This diagram indicates data cleaning procedures leading to final sample used in research study

Participants were Medicaid enrollees with DOBs between Jan 2005 and Nov 2018 and who received an autism diagnosis between Sept 2015 and Feb 2020 (to prevent skew in the data due to the Covid-19 pandemic). In late 2014, Medicaid converted from ICD-9 to ICD-10; to keep the diagnosis as controlled as possible, we only included children diagnosed in ICD-10 system (F84.0 Autistic Disorder). Children with claims data for at least one year prior to autism diagnosis were included in analyses (See Fig. 1 for accounting of data cleaning procedures; *n* = 13,850). Only White and Black identified participants were included in the analyses of the study (*n* = 12,241), as Missouri Medicaid did not provide ethnicity to researchers, and other racial subgroups samples were too small to draw conclusions.

This study explored how multiple elements of a child’s identity (race, sex, disability, and geographical region) may impact their access to and experience within the medical system. Missouri Medicaid covers adults and children who are categorized as low income (i.e., for a household of four, income before taxes below $35,245 qualifies), and those with a disability. Medicaid eligibility was included in initial data, and families that qualified because of a pre-existing disability were excluded.

### Procedure

Data security procedures and protocol were approved by University of Missouri-St. Louis Institutional Review Board. Participants were autistic children diagnosed in the Missouri Medicaid system, and de-identified data was provided to the researchers. To maintain confidentiality, age in weeks at autism diagnosis and prior diagnoses was tabulated before data was received by authors.

### Variables

#### Sociodemographics

Sex and race were collected through Medicaid claims. Geographical status was categorized as rural or urban based on the county code and Missouri Medicaid classifications. Missouri counties are considered rural by Medicaid if there are less than 150 people per square mile and don’t contain a central city in a “Metropolitan Statistical Area.”

#### Behavioral Diagnoses

Missed diagnosis were considered to occur if children in the sample received any diagnosis in ICD-9 or ICD-10 codes other than autism during health visits, within 6 primary categories: ADHD and related disorders, conduct and related disorders (ODD and CD), adjustment disorders, Anxiety-OCD and related disorders, emotion and mood disorders, and intellectual disability Disorders. Diagnostic categories were created from a list of possible Medicaid claims diagnostic codes to represent a breadth of childhood disorders. This diagnostic ordering process was inspired by, but not limited to, those chosen by Mandell et al. (2007). ‘No prior diagnoses’ was also considered a category. We were not able to distinguish between co-occurring diagnoses or incorrect diagnoses.

### Data Analytic Plan

The study was pre-registered at Open Science Framework (Registration DOI Retracted for Blinding). Preliminary analyses checked t-tests, correlations, and ANOVA testing; whether age at diagnosis was related to each of the predicted dummy-coded variables (sex [1 = male], geography [1 = urban], race [1 = White], disability [1 = had Medicaid code of intellectual disability pre-autism diagnosis]).

Aim 1 used a blocked linear regression with sociodemographics and number of prior diagnoses as predictors and age of autism diagnosis as the outcome. For aim 2, the a priori plan was to use a single multinomial logistic regression with the specific prior diagnosis as the outcome, with geography, race, and sex as predictor variables. A large proportion of children had a high diagnostic load (19.1%), and the authors did not want to force children into one diagnostic category, but rather capture their multiple diagnoses. Thus, the plan was switched to running six individual logistic regressions, with post-hoc explorations of multiple overlapping diagnoses. The outcomes in logistic regressions were the presence (1) or absence (0) of the following: ADHD diagnosis, conduct related diagnosis, adjustment and related diagnosis, anxiety and related diagnosis, mood and related diagnosis, and intellectual disability diagnosis. The Benjamini–Hochberg False Discovery Rate (Benjamini-Hochberg, 1995) correction was used to protect against multiple comparisons inflation of type 1 error, adjusting for the 22 regression findings between aims 1 and 2. In exploratory analyses, trends within race, sex, and geographical locale were assessed based on patterns in the descriptive analyses. Chi-square distribution testing with a Benjamini–Hochberg False Discovery Rate corrected significant difference testing was used to assess post hoc analyses.

## Results

### Descriptive Analysis

**Fig. 2:**
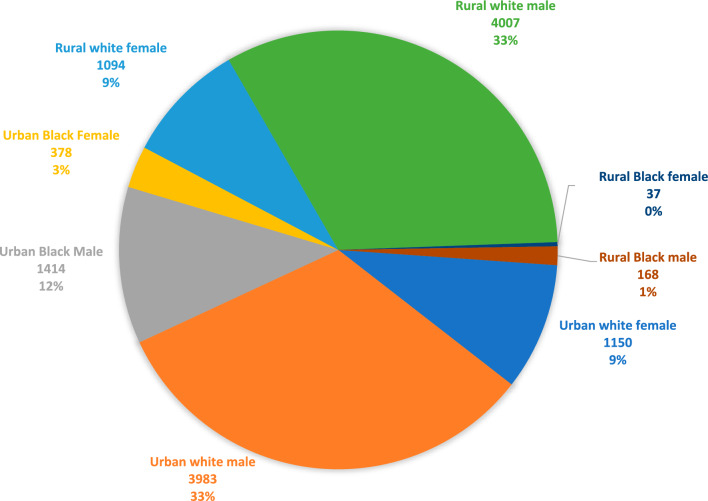
This pie chart shows intersectional identities of autistic Missouri Medicaid children based on included sociodemographic variables

Tables 1 and 2 show descriptive statistics of the sample (*n* = 12,241). The average age of diagnosis was 362.35 weeks (6.9 years), the median was 355 weeks (6.8 years), and the mode was 156 weeks (3.0 years). White children were diagnosed older than Black children (7.05 years old vs. 6.55;* p* < 0.001), and among White participants, no significant differences in average age of diagnosis were found between urban and rural populations (*t*(10,232) = − 1.53, *p* = 0.126, 95% CI [− 11.08, 1.36]; Cohen’s *d* = − 0.030), nor between male and female (*t*(10,240) = 1.21, *p* = 0.225, 95% CI [− 2.86, 12.17]; Cohen’s *d* = − 0.029). Among Black participants, no significant differences in average age of diagnosis were found between urban and rural (*t*(1,996) = 0.05, *p* = 0.959, 95% CI [− 22.099, 23.28]; Cohen’s *d* = 0.004). There were differences based on sex, *t*(1996) = 2.082, *p* = 0.037, 95% CI [1.04,34.89]; Cohen’s *d* = 0.115, such that Black girls were diagnosed significantly younger than Black boys. See Fig. 2, and Supplemental Table 1, for intersectional sociodemographic comparisons.

**Table 1:** Descriptive Statistics of the Autistic Medicaid Sample

Variables	*n*	% or Mean	Range or SD
Average age of Dx with ASD	12,240	362.35	160.15
Sex (Male)	12,241(9581)	78.27	0–1
Race			0–1
White	10,242	83.69%	
Black/African American	1999	16.33%	
Geography (Urban)	12,241(6925)	56.57%	0–1
Pre-diagnostic pictures			
Diagnosed with ADHD and related	3191	26.06%	0–1
Diagnosed with conduct and related	2042	16.68%	0–1
Diagnosed with adjustment and related	785	6.41%	0–1
Diagnosed with anxiety and related	1226	10.00%	0–1
Diagnosed with mood and related	899	7.34%	0–1
Diagnosed with intellectual disability and related	479	3.91%	0–1

**Table 2:** Detailed Descriptives of Autistic Medicaid Sample by Racial Groupings and Sex Groupings

	Racial groups		*p*	Effect size Cohen’s D or Phi	Sex groups		*p*	Effect size Cohen’s D or Phi
Variables	White	Black			Female	Male		
Sex (Male) or race (Black)	78.08%	79.24%	.250	− 0.010	15.6%	16.5%	.250	− 0.010
Geography (Urban)	50.11%	89.65%	< .001	− 0.295	57.4%	56.3%	.319	− 0.009
ASD dx	366.59	340.60	< .001	0.163	357.26	363.76	< .001	− 0.041
Pre-diagnostic pictures								
Diagnosed with ADHD and related	27.45%	18.95%	< .001	0.072	21.42%	27.35%	< .001	0.056
Diagnosed with conduct and related	17.77%	11.11%	< .001	0.066	15.45%	17.02%	.054	0.017
Diagnosed with adjustment and related	6.90%	3.91%	< .001	0.045	6.95%	0.00%	.197	− 0.012
Diagnosed with anxiety and related	11.07%	4.60%	< .001	0.080	11.01%	9.74%	.052	− 0.018
Diagnosed with mood and related	7.74%	5.30%	< .001	0.035	7.41%	7.32%	.890	− 0.001
Diagnosed with intellectual disability and related	3.87%	4.10%	.634	− 0.004	5.00%	3.61%	.001	− 0.030

Significance based on *p* value from a chi square or t test. DF for all chi square = 1, for T test, 12,238. To interpret phi, a small effect size is .1 or less, medium is .3, and .5 is a large effect. For Cohen’s d, .2 or under is considered to be a small effect size

Overall, 36.11% had prior diagnosis of any kind (*n* = 4,420); in particular, 16.87% (2066) had one prior diagnosis, and 0.12% had all six categories of prior diagnoses. The most common overlap was ADHD and Conduct and related disorders, where 69.4% of children that had ADHD also had conduct disorder. The second most common overlap was ADHD and anxiety (41.7%).

### Main Analysis

#### Age of Autism Diagnosis

A two-block hierarchical multiple regression was conducted to measure the impact of race, sex, geographical status, and in the second block, number of prior diagnoses, on age of diagnosis **(**Table 3). Overall, the final model explained 15.9% of the variance; being White, living urban, and having more prior diagnoses was associated with older age of autism diagnosis.

**Table 3:** Linear Regression with Outcome of Age of Diagnosis

Predictor	Unstandardized B	Unstandardized S.E	Standardized Beta	T	Significance	95% Confidence Interval
Block 1 model						
Race (White = 1)	15.13	3.77	0.04	4.01	< .001	[7.733, 22.523]
Sex (Male = 1)	4.49	3.22	0.01	1.39	0.164	[− 1.827, 10.803]
Geographic (Urban = 1)	9.49	2.81	0.03	3.38	< .001	[3.983, 14.991]
Block 2 model						
Race (White = 1)	15.13	3.77	.04	4.01	< .001	[7.73, 22.52]
Sex (Male = 1)	4.49	3.22	.01	1.39	.164	[− 1.83, 10.80]
Geographic (Urban = 1)	9.49	2.81	.03	3.38	< .001	[3.98, 14.99]
Any prior diagnoses cumulative	54.86	1.16	0.40	47.43	< .001	[52.597, 57.132]

*N* = 12,230. Block 1 Model: F(3, 12,229) = 16.72,* p* < .001,: r squared block 1 = .004. Block 2 Model: F(4, 12,229) = 577.253, *p* < .001,: r squared block 2 = .159. The Benjamini–Hochberg False Discovery Rate (Benjamini-Hochberg, 1995) correction was used to protect against multiple comparisons inflation of type 1 error, all findings remain significant

#### Examining Prior Diagnoses

A series of logistic regressions (Table 4) were run to examine likelihood of receiving particular diagnoses prior to autism diagnosis.

**Table 4:** Logistic Regressions

Predictor	B	S.E	Wald	df	Significance	Exp (B)	95% CI
ADHD model							
Race (White = 1)	0.44	0.06	47.83	1	< .001	1.55	[1.37, 1.76]
Sex (Male = 1)	0.33	0.05	38.60	1	< .001	1.39	[1.25, 1.54]
Geographic (Urban = 1)	− 0.11	0.04	6.75	1	0.009	0.89	[0.82, 0.97]
Conduct and related Model							
Race (White = 1)	0.44	0.08	31.23	1	< .001	1.55	[1.33, 1.81]
Sex (Male = 1)	0.12	0.06	3.80	1	0.051	1.13	[0.99, 1.27]
Geographic (Urban = 1)	− 0.27	0.05	28.33	1	< .001	0.76	[0.69, 0.84]
Adjustment and related Model							
Race (White = 1)	0.56	0.13	20.08	1	< .001	1.76	[1.37, 2.25]
Sex (Male = 1)	− 0.11	0.09	1.55	1	0.213	0.90	[0.76, 1.06]
Geographic (Urban = 1)	− 0.09	0.08	1.46	1	0.228	0.91	[0.79, 1.06]
Anxiety and related Model							
Race (White = 1)	0.91	0.11	64.01	1	< .001	2.49	[1.99, 3.12]
Sex (Male = 1)	− 0.13	0.07	3.52	1	0.061	0.88	[0.76, 1.01]
Geographic (Urban = 1)	− 0.08	0.06	1.73	1	0.189	0.92	[0.82, 1.04]
Mood and related model							
Race (White = 1)	0.37	0.11	11.31	1	< .001	1.45	[1.17, 1.80]
Sex (Male = 1)	− 0.01	0.08	0.01	1	0.916	0.99	[0.84, 1.17]
Geographic (Urban = 1)	− 0.08	0.07	1.311	1	0.252	0.92	[0.80, 1.06]
Intellectual disability Model							
Race (White = 1)	− 0.17	0.13	1.76	1	0.185	0.84	[0.65, 1.09]
Sex (Male = 1)	− 0.35	0.11	11.01	1	< .001	0.71	[0.58, 0.87]
Geographic (Urban = 1)	− 0.26	0.10	6.90	1	0.009	0.77	[0.64, 0.94]

*N* = 12,231; ADHD Model =  − 2 Log likelihood = 13,925.69, Nagelkerke R Square = 0.013; Conduct and Related Model =  − 2 Log likelihood = 10,942.27, Nagelkerke R Square = 0.012; Adjustment and Related Disorders =  − 2 Log likelihood = 5798.65, Nagelkerke R Square = 0.007; Anxiety and Related Model = 2 Log likelihood = 7863.631, Nagelkerke R Square = 0.016; Mood and Related Model =  − 2 Log likelihood = 6406.707, Nagelkerke R Square = 0.003; Intellectual Disability Model =  − 2 Log likelihood = 4019.122, Nagelkerke R Square = 0.005

##### ADHD

Contrary to hypotheses, being White was associated with being more likely to receive an ADHD diagnosis. Consistent with hypotheses, being male and living in a more rural area were associated with a child being more likely of receiving an ADHD diagnosis. In particular, being White was associated with a child being 1.55 times more likely to be diagnosed compared to being Black; being male (instead of female) 1.38 times more likely to be diagnosed with ADHD.

##### Conduct

Contrary to hypotheses, being White and living rurally were both associated with a child being more likely to be diagnosed with conduct disorders. Being White was associated with a 1.55 times higher likelihood, while living in a rural area left a child 0.24 times (Exp (B) = 0.76) less likely to receive this diagnosis. The findings on sex were not statistically significant.

##### Adjustment

Contrary to hypotheses, being White was associated with a 1.76 times higher likelihood of being diagnosed with adjustment disorders. Sex and geographic region were not significantly related.

##### Anxiety

Being White was associated with 2.49 times greater odds of an anxiety diagnosis. Sex and geographic region were not significantly related.

##### Mood and Related Model

Being White was associated with a 1.45 times more likely chance of being diagnosed with a mood disorder. Sex and geographic region were not significantly related.

##### Intellectual Disability Model

Consistent with hypotheses, being female and living in a rural area were associated with a child being more likely to be diagnosed with an intellectual disability. Boys were 0.24 times (Exp (B) = 0.76) less likely to receive this diagnosis, while those living in urban settings were 0.23 (Exp (B) = 0.77) times less likely. Race was not significantly associated.

### Post-hoc Exploratory Analyses

**Fig. 3:**
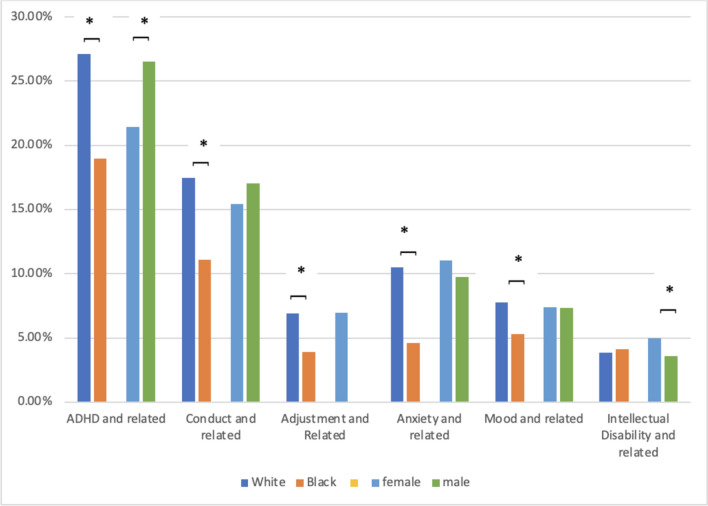
This bar chart shows the percent of autistic children on Missouri Medicaid with each examined prior diagnosis by race and sex. Note: * represents a significant difference at *p* < .05

Post-hoc and exploratory analyses were initiated to investigate findings in cases that violated *apriori* predictions (Fig. 3). Urban White females and males appear similar with prior diagnostic rates of 36.34% and 36.55%, but look different than Urban Black populations, where males had a prior diagnostic rate of 27.86% and females had a prior diagnostic rate of 20.63%. Both White males and females had significantly more prior diagnoses than their counterparts, Black males, *X*^2^ (1) = 34.99, *p* < 0.001, phi = 0.081, and Black females, *X*^2^ (1) = 29.83, *p* < 0.001, phi = 0.14, respectively.

The differences in conduct disorders were more closely examined in the urban subsample because they violated apriori hypotheses. Rates of conduct disorders in Black and White children in urban samples were significantly different, *X*^2^ (1) = 32.59, *p* < 0.001, phi = 0.093, a small effect size, with White children having higher rates of diagnosis than Black children. In the urban male sample, racial differences in diagnostic rates of conduct disorder were significant, *X*^2^ (1) = 22.96, *p* < 0.001, phi = 0.065, a small effect size, with White males being diagnosed at significantly higher rates than Black males. The differences in ADHD were similarly examined because they impacted a large segment of the participants and were contrary to hypotheses, with White males having significantly higher rates than Black males.

## Discussion

This project examines how the aspects of the identities of autistic participants impacted their diagnostic pathways, via age of autism diagnosis and prior comorbidities. The data showed that being Black was associated with a younger age of diagnosis (around 6 months younger), contrary to hypotheses. Jo et al. (2015) have similar findings; White children had an older age of diagnosis compared to peers with minoritized racial backgrounds. The authors (post-analysis) theorized that this could be because children with subtle presentations, who are also of a minoritized racial background, might be under-represented. Another possibility is that White children are getting more diagnoses overall due to increased access to care.

It is important to note that Black children only represent 14.43% of our sample. In 2017 (the midway point of the autism diagnosis data), of the Black and White children on Medicaid in our age range, 71.7% were White, and 28.29% were Black (*numbers provided *via* communication with Missouri Medicaid*). Regarding gender, the same year, children on Medicaid under 10 years old the population was 48.7% female. Thus, *Black children and girls are both under-represented in our subsample* of autistic children compared to the Medicaid sample. It is likely that Black children and girls with more clear presentations, and parents motivated to attain an early diagnosis are included, while those with more complex presentations, either due to intellectual disability or strong cognitive and language ability, are not diagnosed and included in our data.

The findings for age of diagnosis and sex were not significant; but girls were more likely to be diagnosed with intellectual disability, consistent with the extant literature (Jeste & Geschwind, 2014). This further points to an under-representation of girls with subtle presentations of autism, with girls only representing 21.84% of our sample. Living in an urban area was associated with an older age of diagnosis, contrary to hypotheses. Though rural and urban differences in prevalence have not been thoroughly explored, extant literature suggests barriers for rural families, making this finding rather surprising (Antezana et al., 2017; Ning et al., 2019). Rural children were more likely to be diagnosed with intellectual disability. It may be the case that rural children are also underrepresented where symptoms are more subtle. The overlap between race and urbanicity is not to be ignored in this dataset, where less than two percent of the sample were both Black and living in rural areas.

The average age of autism diagnosis was almost 7 years old, about 3 years older than national averages (Maenner et al., 2023), however, the modal age, around 3, is closer to an age for early interventions. This may suggest that less progress has been made in early identification for low-income populations, such as this Medicaid sample, compared to the wider population.

### Prior Diagnoses

Having no prior diagnoses before the autism diagnosis was associated with being Black and living in an urban area. More prior diagnoses were associated with later autism diagnosis. Increased diagnostic load is likely an indicator of a complex presentation (Casanova et al., 2020). It could also be related to access to medical care, with more diagnoses reflecting more visits to doctor’s offices, and referrals or medical orders in the healthcare system. Race was a significant predictor of being diagnosed with every prior diagnosis, aside from intellectual disability, likely indicating more diagnostically complex autistic Black children are not included in this sample as they are not yet diagnosed with autism, hence the under-representation by racial group noted earlier. This might reflect how providers view children, as well as beliefs families hold about child development (i.e., less diagnostic-seeking behaviors at early signs, more tolerance for variability in child development).

Being White was associated with having an anxiety disorder, adjustment disorder, and mood disorder. Broder-Fingert et al. (2013) found racial differences in treatment of autistic children, specifically, Black children had fewer appointments and evaluations from psychiatrists. While autism is considered primarily biological in nature (Parellada et al., 2014), anxiety and mood disorders feature a combination of environmental and biological precedents (Schiele & Domshke, 2018). One explanation is that Black families are a protective factor for neurodiverse children, indeed, many protective factors have been hypothesized through cultural and family structures including resilience, social support, feelings of efficacy, extended family network support and more (Liu et al., 2017; Taylor et al., 2015). Again, the under-representation of Black children in the sample complicates interpretations of this finding.

Similar rates of ADHD co-diagnosis (24%) as Rosen and colleagues were found (30%; 2018). In the ADHD model, children who were White, males, and living rurally were all more likely to be diagnosed with ADHD. Significant research supports racial disparities in diagnosis of ADHD, wherein Black children are diagnosed less despite similar symptoms (Winders-Davis et al., 2021). ADHD is commonly diagnosed by pediatricians or family practitioners, who children living rurally have more access to (Committee on Quality Improvement, Subcommittee on Attention-Deficit/Hyperactivity Disorder, 2000). Similarly, being white and living rurally were both related to being diagnosed with conduct disorder. This could be because school providers are more comfortable offering this diagnosis (then reported to family practitioners, entering the medical record to support prescriptions or referrals). Boys and girls did not have different rates of diagnosis, in line with prior research which indicates rates of conduct disorders are similar across genders (Rowe et al., 2010).

The relationships among race, sex, and geographic region in ADHD and conduct disorders warranted post-hoc analyses. When looking at urban males, White boys had significantly more diagnoses than Black boys, and the pattern held with urban females. Prior research has found disparities in ADHD, with Black children having significantly lower rates of diagnosis and medication (Coker et al., 2016; Shi et al., 2021; Winders Davis et al., 2021). While biological/genetic indicators may cause disparities between male and female prevalence (Merikangas & Almasy, 2020), racial disparities are hypothesized to be influenced by environmental factors such as provider bias or structural racism (Moody, 2016; Winders Davis et al., 2021). This could include parent beliefs about symptoms, cultural influences on co-occurrence (Slobodin & Masalha, 2020), or cultural insensitivity in diagnostic processes and racism in schools (Kang & Harvey, 2020; Moody, 2016). These same factors may have influenced the under-representation of Black children in this Medicaid sample.

The data on anxiety, mood, and adjustment disorders have many similarities. Our study found lower rates of anxiety co-occurrence (around 10%) than is established in the literature (Lai et al., 2019; Rosen et al., 2018). In the model of anxiety disorders, sex was not a significant predictor, so *apriori* hypotheses were not supported. That being said, Lai et al. (2011), also found similar rates of anxiety, depression, and OCD symptoms in male and female autistic participants, so there is some precedent in the literature for these findings.

Mandell et al. (2007), had very different findings. The prior study was conducted over 25 years ago, in an urban center on the east coast, with no analysis of geographical influences and limited gender analysis. It categorized children in the prior diagnosis they had received the most, rather than across multiple diagnostic categories, and found that Black children were more likely to be diagnosed with another condition before autism. There are a few reasons why our findings may not match the earlier data. First, autism diagnostic rates in general, and in particular among people of color, have increased dramatically (Nevison & Zahorodny, 2019). The average age of diagnosis has gotten younger, while the DSM-5 put autism on a spectrum, meaning that a broader group of people fall under the umbrella (APA, 2013). Changes to the healthcare system mean that more people have healthcare in the US as compared to the 1990s, allowing for more access to services. This paper’s significant updates to prior findings highlight the importance of replication in the field of clinical psychological research (Fletcher, 2021).

Intellectual disability diagnostic rates in this data (3.7%) were much lower than they are hypothesized to be, which is around 36% (Maenner et al., 2023). Children may be carrying ‘developmental delay’, or other similar terms, in medical records, and will later be diagnosed with intellectual disability. Furthermore, children who were brought on Medicaid with a diagnosis of intellectual disability may have been cut during data cleaning procedures which excluded participants who came on Medicaid due to a disability.

## Limitations

One limitation is that the authors cannot speak to accuracy or severity of diagnoses; they could be accurate co-occurring conditions or better explained by autism. Fombonne et al. (2004) showed that medical records were highly accurate in autism diagnoses, but Medicaid claims are not a perfect replication of medical records. Inconsistent coverage can lead to impacts on claims data and health effects for enrollees (Bensken et al., 2022). Relatedly, there is an underrepresentation in our data of Black children and girls, as well as likely an undercounting of intellectual disability.

Although Medicaid data facilitates sampling a large, diverse group, there are also people excluded. One large limitation is that our data did not contain ethnicity, and some racial groups had to be cut due to numbers too small to interpret (especially when subdivided by gender).

Further, making geographical regions into a dichotomous variable collapsed significant variability within urban and rural labels, for example how well-resourced an area is, how populated, and racial and socioeconomic differences within urban or rural areas. All dichotomized variables (race, rurality, diagnostic status, and gender) prevented more nuanced and complex examination of the influences of these variables through clustering or other analysis. This study, despite its high power and strong sample size, showed small effect sizes. This likely means that there are many other factors which influence these outcomes.

## Future Directions

There is much more to be done in terms of the examination of racism, sexism and ableism in neurodevelopmental disorders and co-occurring conditions. An investigation into what other factors influence age of diagnosis and prior diagnostic picture, including social support to the family, early childhood care centers, screeners used in primary care office, number of early childhood pediatric well-child visits, would be recommended.

Another important direction is related to access. While Medicaid, by its very existence, increases access to healthcare, the picture is more complex. Other barriers to access include hours of operation of doctors’ offices, parents’ literacy, distance to doctor or pharmacy, coverage and availability of certain prescriptions, availability of providers and waitlists, language barriers, provider bias, family comfort, treatment by practitioners, and more. Urbanicity is limited in its measure of access, as some urban centers in Missouri may have fewer providers per capita and longer waitlists than rural areas.

## Supplementary Information

Below is the link to the electronic supplementary material.

Supplementary file1 (DOCX 13 kb)
