# Supplementary material for: Prior Diagnoses and Age of Diagnosis in Children Later Diagnosed with Autism
Source: J Autism Dev Disord. 2024 Nov 25;56(4):1460–72. doi: 10.1007/s10803-024-06637-3 (PMC12987802; doi:10.1007/s10803-024-06637-3)
Supplement: Supplementary file 1 — Supplementary file1 (DOCX 13 kb) [file 10803_2024_6637_MOESM1_ESM.docx]

**Supplemental Table 1** Intersectional Analyses

| Sub-categories | % within group prior dx | N | Average age of ASD dx | Any previous dx |
| --- | --- | --- | --- | --- |
| Urban White female | 36.34% | 1150 | 365.52 | 418 |
| Urban White male | 36.55% | 3983 | 369.96 | 1456 |
| Urban Black male | 27.86% | 1414 | 345.41 | 394 |
| Urban Black female | 20.63% | 378 | 321.94 | 78 |
| Rural White female | 37.56% | 1094 | 360.23 | 411 |
| Rural White male | 36.33% | 4007 | 365.16 | 1456 |
| Rural Black female | 51.35% | 37 | 372.78 | 19 |
| Rural Black male | 29.76% | 168 | 334.26 | 50 |

Total N: 12,241
